# Anatomical and biochemical studies of *Spartium junceum* infected by *Xylella fastidiosa* subsp. *multiplex* ST 87

**DOI:** 10.1007/s00709-021-01640-2

**Published:** 2021-04-15

**Authors:** S. Falsini, C. Tani, G. Sambuco, A. Papini, P. Faraoni, S. Campigli, L. Ghelardini, G. Bleve, D. Rizzo, M. Ricciolini, I. Scarpelli, L. Drosera, A. Gnerucci, F. Peduto Hand, G. Marchi, S. Schiff

**Affiliations:** 1 Dipartimento di Biologia, Università degli studi di Firenze, via P.A. Micheli 3, 50121 Firenze, Italy; 2 Dipartimento di Scienze Biomediche, Sperimentali e Cliniche, Università degli Studi di Firenze, viale G. Pieraccini 6, 50139 Firenze, Italy; 3 Dipartimento di Scienze delle Produzioni Agroalimentari e dell’Ambiente, Università degli Studi di Firenze, Piazzale delle Cascine 28, 50100 Firenze, Italy; 4 Istituto di Scienze delle Produzioni Alimentari, Consiglio Nazionale delle Ricerche, Lecce, Italy; 5 Regione Toscana, Servizio Fitosanitario Regionale e di Vigilanza e Controllo Agroforestale, Via A. Manzoni 16, 50121 Firenze, Italy; 6 Dipartimento di Fisica e Astronomia, Università di Firenze, Via Sansone 1, 50019 Sesto Fiorentino, (FI) Italy; 7 Department of Plant Pathology, Ohio State University, Columbus, OH 43220 USA

**Keywords:** *Spartium junceum*, *Xylella fastidiosa* subsp. *multiplex ST 87*, Light microscopy, SEM, Anatomy, Interaction

## Abstract

*Spartium junceum* L. is a typical species of Mediterranean shrubland areas, also grown in gardens and parks as an ornamental. In recent years in Europe, *S. junceum* has been recurrently found to be infected by different subspecies and genotypes of the quarantine regulated bacterium *Xylella fastidiosa* (Xf). This work presents for the first time the anatomy of *S. junceum* plants that we found, by means of genetic and immunochemistry analysis, to be naturally infected by Xf subsp. *multiplex* ST87 (XfmST87) in Monte Argentario (Grosseto, Tuscany, Italy), a new outbreak area within the EU. Our anatomical observations showed that bacteria colonized exclusively the xylem conductive elements and moved horizontally to adjacent vessels through pits. Interestingly, a pink/violet matrix was observed with Toluidine blue staining in infected conduits indicating a high content of acidic polysaccharides. In particular, when this pink-staining matrix was observed, bacterial cells were either absent or degenerated, suggesting that the matrix was produced by the host plant as a defense response against bacterial spread. In addition, a blue-staining phenolic material was found in the vessels and, at high concentration, in the pits and inter-vessels. SEM micrographs confirmed that polysaccharide and phenolic components showed different structures, which appear to be related to two different morphologies: fibrillary and granular, respectively. Moreover, our LM observations revealed bacterial infection in xylem conductive elements of green shoots and leaves only, and not in those of other plant organs such as roots and flowers.

**Supplementary Information:**

The online version contains supplementary material available at 10.1007/s00709-021-01640-2.

## Introduction

*Xylella fastidiosa* (Wells et al. 1987) (Xf) is a Gram-negative bacterium, which presents six subspecies: *fastidiosa*, *multiplex*, *sandyi*, *morus*, *tashke*, and *pauca* (EFSA 2020)*.* It is the causal agent of *Pierce’s disease* in grapevine, the *olive quick decline syndrome* in olive, the *citrus variegated chlorosis* in citrus plants, and *leaf scorch disease* in almond, coffee, oleander, elm, sycamore, pecan, pear, mulberry, maple, and oak (Hopkins and Purcell 2002; Janse and Obradovic 2010; Purcell 2013). Xf is always associated with its plant host or its insect vectors, which are sharpshooter leafhoppers (Hemiptera: Cicadellidae: Cicadellinae), spittlebugs (Hemiptera: Cercopidae) (Severin 1949; Severin 1950), and cicadas (Hemiptera: Cicadidae) (Paião et al. 2002); no evidence has been reported of Xf free-living outside its hosts. In plants, Xf lives only in xylem vessels while in the hemipteran insects, it colonizes the cuticular surface of the oral cavity or rather the anterior foregut (Backus and Morgan 2011). The similarity between the two exclusive niches of Xf (i.e., host plant and insect vector) is that in both cases, the colonized tissues are composed of non-living cells with which the bacterium mainly interacts during its life cycle. This phenomenon is probably due to the Xf genome adaptation. The genome of Xf shares some similarities with that of xanthomonads, typical bacterial pathogens, but in contrast, it has a much-reduced genome and lacks a Type III secretion system (T3SS) (Simpson et al. 2000). T3SS are complex bacterial structures that are delivered in living tissues to suppress the immune response of the host; the lack of this system may explain why Xf survives only when it is surrounded by dead cells (Roper et al. 2019).

It is well established that after colonizing xylem conduits, Xf leads to occlusion of the vascular system due to gums, tyloses, and bacterial biofilms (Sun et al. 2013; Pèrez-Donoso et al. 2016). Depending on the extent of the occlusion, vascular plugs cause partial or total arrest of water flow from the roots to the leaves. Thus, typical symptoms of the disease are leaf scorching at the early stages followed by withering of shoots until plant death, which happens in a few years (Saponari et al. 2013).

The presence of Xf has been documented in several plant species of commercial interest especially in Italy, where it caused the death of thousands of centenarian olive trees (Cariddi et al. 2014). However, many aspects of the disease still need to be clarified. Although the transmission of Xf through the hemiptera insects is well documented, the mechanism of pathogenesis is still under investigation due to the complexity of the infection process (Cornara et al. 2016; Novelli et al. 2019).

Among the several diseases caused by the different subspecies of Xf, the present work is focused on the infection of *Spartium junceum* L. (Fabaceae) (Spanish broom) by *X. fastidiosa* subsp. *multiplex* (Xfm). Although Xfm is thought to be native of North America, this subspecies was first detected in 1935 in Argentina causing plum leaf scald and then in 1978 in Paraguay and Brazil (Nunes et al. 2003). More recently, 41 species of angiosperms have been found susceptible to Xfm in Europe (EU 2019; Landa et al. 2020).

*S. junceum* is an indigenous species of the Mediterranean basin and it plays an important role in the coastal Tuscan landscape. Because of its marked adaptability and high resistance to drought, this species has also been successfully used to protect and colonize slopes that were subject to superficial erosion phenomena (Preti and Giadrossich 2009). Historically, the plant was cultivated and used as a raw textile material to make ropes spun for vessels and shipbuilding, as well as nets, bags, and sails (Katović et al. 2012). Recently, a medieval shipwreck excavation found galleys from the Byzantine period in which treenails were mainly constructed with Spanish broom wood (Akkemik and Kocaba 2012). *S. junceum* is also known for its medicinal properties due to anti-neoplastic activity (Abusamra et al. 2015), and as an ornamental and also poisonous plant due to the presence of quinolizidine alkaloids (Giménez et al. 2017).

During the execution of an early detection surveillance program for Xf, the DNA of Xfm was first detected in a plant of *S. junceum* growing in the municipality of Monte Argentario (Grosseto, Tuscany, Italy) in October 2018 (Marchi et al. 2018). Soon after the presence of its DNA was detected in some other hosts, the bacterium was isolated, a novel sequence type (ST87) was identified, and three genomes were deposited in the GenBank database (Saponari et al. 2019; Giampetruzzi et al. 2019).

The present research aimed to describe the anatomy of *S. junceum* plants naturally infected by Xfm ST87, highlighting pathological characteristics and possible diagnostic anatomical features. For this purpose, we investigated the interaction of Xfm ST87 with *S. junceum* tissues and the mechanism of bacterial spread through the plant xylem vessels until the formation of vascular plugs. Additionally, we investigated the nature of the gel matrix associated with the pathogen in xylem vessels, which could be linked to the complex mechanism of plant defense response to this parasite.

## Materials and methods

### Plant sampling and indexing for Xf

After the first detection of Xfm ST87 in October 2018, demarcated areas were established according to EU Decision 2015/789, and monitoring, plant sampling, whole nucleic acid extractions, and molecular indexing for Xf were carried out according to PM 7/24-3 unless otherwise stated (EPPO 2018). Briefly, during inspection procedures, specimens of *S. junceum* plants were collected and selected mostly based on the presence of scorching of green shoots, a nonspecific symptom putatively ascribable also to Xf. Samples were immediately stored at 5 °C for up to 2 days prior to processing. Whole nucleic acids were extracted from green shoots using the CTAB-based protocol and stored at − 20 °C until further use. Initial screening for the presence of Xf was carried out by at least two independent real-time (qPCR) protocols (Harper et al. 2010; Francis et al. 2006; Ouyang et al. 2013). A selection of samples found to be infected by Xf according to qPCR results were further subject to amplification and sequencing of either the sole *nuoL* gene fragment or all the 7 genes (*cysG*, *gltT*, *holC*, *malF*, *leuA*, *nuoL*, and *petC*) required by the MLST-typing approach (Yuan et al. 2010). For qPCR experiments, the Quantinova Probe PCR kit (Qiagen, Hilden, Germany) and Quantinova SYBR Green PCR Kit (Qiagen, Hilden, Germany) master mixes were used; meanwhile for conventional PCR, the GoTaq G2 polymerase (Promega) was used. All conventional PCR products were visualized after electrophoresis in 1.5% agarose (Genaxxon) gels in 1 × Tris–acetate–EDTA buffer (Invitrogen) and staining with Midori Green (Nippongenetics; 0.05 μl mL^−1^). They were purified using FastAP and Exonuclease I (Thermo) and Sanger sequenced. Sense and antisense nucleotide sequences were visualized and checked for quality using CHROMAS LITE 2.01 (Technelysium), aligned using MUSCLE as implemented in MEGA6 (Tamura et al. 2013), and single consensus sequences were determined. Searches were carried out on the Xf MLST database website (http://pubmlst.org/xfastidiosa) to determine the allele and the resulting sequence type (ST).

### *X. fastidiosa* isolation and characterization

Xf isolation was performed at different time points throughout 2019 from plants previously indexed for the presence of Xf (see above). Green shoots were cut to a length of approximately 10 cm with flame-disinfected shears, surface disinfected by immersion in 80% ethanol followed by flaming, and finally cut into fragments of approximately 3 cm in length inside a laminar flow hood. Occasionally, bacterial isolation from different plant parts was also tested. Isolations were attempted on BCYE agar as described in Bleve et al. (2016) using 40 fragments from each plant and by delivering a single blot of sap/fragment into the isolation plate (8 fragments/plate). Plates were incubated at 28 °C and checked every 2 days during the first week of incubation and once a week thereafter for a total of 8 weeks. Plates containing bacterial colonies that become visible to the unaided eye within the first 72 h of incubation were discarded; meanwhile, those that became visible thereafter were streaked twice for purity on BCYE agar, suspended in sterile distilled water, and identified as Xf based on the results of qPCR (Harper et al. 2010) performed on 1 μl of bacterial suspension after boiling for 10 min. The DNA of two Xf isolates from each plant was extracted using CTAB buffer, resuspended in Tris–HCl 10 mM pH8, and further characterized to subspecies and ST level using the MLST approach using the conditions described by Yuan et al. (2010). QuantiFast Multiplex PCR Master Mix (Qiagen) and GoTaq G2 polymerase (Promega) were used for qPCR and conventional PCR, respectively. qPCR conditions and MLST gene fragments amplification, purification, and analysis were performed as described above. All isolation procedures were carried out in the laboratory of the Phytosanitary Service of the Tuscany Region in Porto Santo Stefano (Grosseto, Italy).

### *Spartium junceum* sampling

Primary roots, branches, apical, medial, and basal portions of green shoots, leaves, and flowers were collected from three different *S. junceum* plants (replicates), hereafter referred to as SJ 668, SJ 911, and SJ 796, from which Xfm ST87 had been previously isolated. SJ 668 and SJ 911 plants showed scattered desiccation of shoots while SJ 796 appeared to be asymptomatic. Roots, twigs, and green shoots were collected from SJ 668 in October 2019, before its eradication according to EU Decision 2015/789, and from SJ 911 in February 2020. True leaves, green shoots, and flowers were collected from SJ 796 in May 2020. A fourth *S. junceum* plant, SJ 777, that had tested negative to qPCR indexing for Xf and from which the bacterium could not be isolated on BCYE, was collected in the same area in May 2020 and used as negative control. Finally, a healthy *S. junceum* plant was collected in an area close to Ginestra Fiorentina in the province of Florence (Tuscany, Italy), approximately 160 km away from the infected area, where there is no evidence of Xf presence, and used as naïve control.

All plant material used in this work was homogeneous in terms of region of origin, environmental conditions of growth, and age (3–4 years).

### Light, immunohistochemistry, and scanning electron microscopy

For light microscopy (LM) observations, samples were fixed with FAA (5:5:90 v/v/v 40% formaldehyde: glacial acetic acid: 70% ethanol) at 5 °C and then dehydrated in ethanol series (70%, 80%, 95%, and 100% v/v). These samples were then embedded in Technovit 7100 resin and sectioned with a Reichert-Jung Ultracut E microtome. Primary shoots (approximately 0.5 cm in diameter), older branches, leaves, flowers, and roots were sectioned with a microtome to obtain 2 μm sections. Transverse or longitudinal sections were mounted on microscope slides and stained using different protocols: 0.5% Toluidine blue O in 0.1% Na_2_CO_3_ at pH 11.1 (O’Brian and McCully 1981), which at this pH, stains acidic polysaccharides pink/violet and phenolic components blue (Dong et al. 1997; Tagne et al. 2002; Crews et al. 2003); Alcian Blue for acid mucopolysaccarides; and Periodic acid-Schiff staining (PAS) reaction for polysaccharides. A Leitz DM-RB Fluo Optic microscope (Wetzler, Germany) equipped with a digital camera Nikon DS-L1 (Tokyo, Japan) was used for microscopic observations.

Transversal and longitudinal sections 2-μm-thick samples sections, embedded in Technovit 7100 resin, as well as 20-μm-thick sections fixed in FAA and maintained in 50% ethanol, were used for immunofluorescence determination. In order to remove the resin and retrieve the antigen, the embedded sections were treated with 10% H_2_0_2_ or a saturated NaOH solution diluted 1:2 in ethanol for 15′ and then washed in distilled water. For the immunohistochemical assay, the Immunofluorescence kit IF 07419 by Loewe® Biochemica GmbH (Sauerlach, Germany) was used. After a wash in PBS, the primary antibody against Xf diluted 1:1000 in PBS was applied to each slide incubated for 30 min in a humid chamber at room temperature and then washed twice for 7 min with 0.1% Tween 20 in PBS. Finally, FITC-conjugated goat anti-rabbit secondary antibody diluted 1:150 in PBS was applied and incubated for 30 min in a humid chamber at room temperature, After incubation, the slides were rinsed as previously described and mounted with Fluoroshield with DAPI (F6057, Merck, Germany). Slide observations were made using a Nikon inverted microscope (Nikon Eclipse Ti) and the following epifluorescence filter sets were used: excitation 365 nm, emission 400 nm hi-pass to observe DAPI staining; excitation 485 nm, emission 524 nm for FITC fluorescence staining, and excitation 575 nm, emission 661 nm for red fluorescence (lignin fluorescence). Images were acquired with the Coolsnap HQ2 CCD camera (Princeton instruments, USA).

For scanning electron microscopy (SEM) studies, samples were fixed with FAA and then processed with the immersion for 24 h each in a series of increasing concentrations of ethanol (70%, 80%, 95%, and 100% v/v). After dehydration, samples were critical point dried and gold metalized using a sputter coater. Finally, samples were observed by SEM Zeiss EVO 40 at MEMA center of the University of Florence (Centro di Servizi di Microscopia Elettronica e Microanalisi—MEMA). The instrument was set at 15 kV and at different working distances ranging from 6.0 to 5.5 mm.

## Results and discussion

### *X. fastidiosa* incidence in *S. junceum*, isolation, and characterization

Between October 2018 and December 2019, a total of 669 *S. junceum* plants were sampled as part of the Xf monitoring program in the demarcated area of Monte Argentario (Grosseto, Tuscany, Italy). According to the results of two independent qPCR specific assays, 106 plants were found to be infected by Xf. To confirm these results, a selection of 70 Xf-positive extracts were subject to amplification of a fragment of the Xf *nuoL* gene. Fifty-five of them yielded an amplicon of the expected size (557 bp) and 51 out of 55 were successfully sequenced in both forward and reverse. Sequence analysis against the Xf MLST database indicated that allele 21 of the Xfm ST87 *nuoL* gene had been sequenced in all samples. MLST analysis was completed on 19 of the 51 *S. junceum* extracts, and the presence of the sole ST 87 [5 (*leuA*), 3 (*petC*), 5 (*malF*), 3 (*cysG*), 3 (*holC*), 21 (*nuoL*), 3 (*gltT*)] was found.

**Fig. 1:**
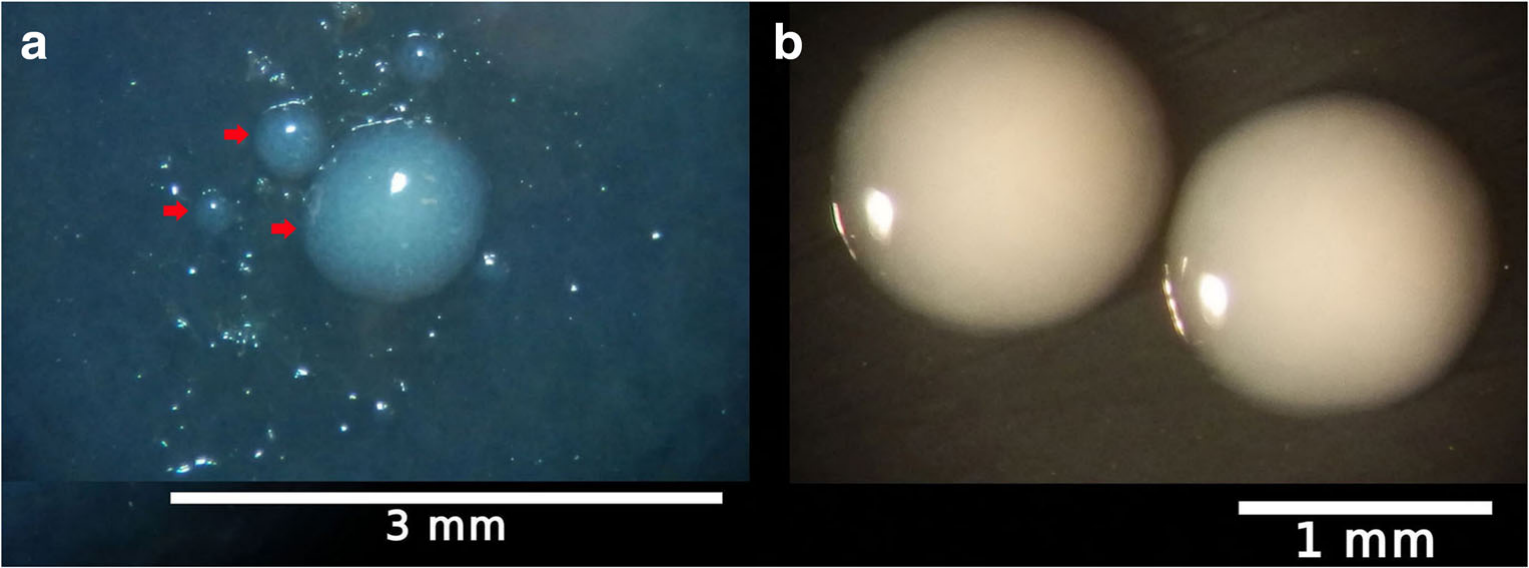
**a** Thirty days old white punctiform bacterial colonies (red arrows) growing on BCYE agar plates within the area of a sap blot squeezed from a fragment of *S. junceum* green shoot (SJ 668); **b** two purified colonies of Xfm ST87 after incubation at 28 °C for 38 days on BCYE agar plates

Bacterial isolation was attempted from 11 *S. junceum* plants arbitrarily selected among those found to be infected according to qPCR and MLST results. Fast growing, often pigmented bacterial colonies usually appeared within the sap blotted areas on BCYE agar during the first 7 days of incubation at 28 °C. Their incidence was low, ranging between 0 and 5% of the analyzed plant fragments, and their DNA could not be amplified with the Xf-specific qPCR protocol (Harper et al. 2010). White, punctiform colonies became visible to the unaided eye usually after 2–3 weeks of incubation (Fig. 1a).

**Fig. 2:**
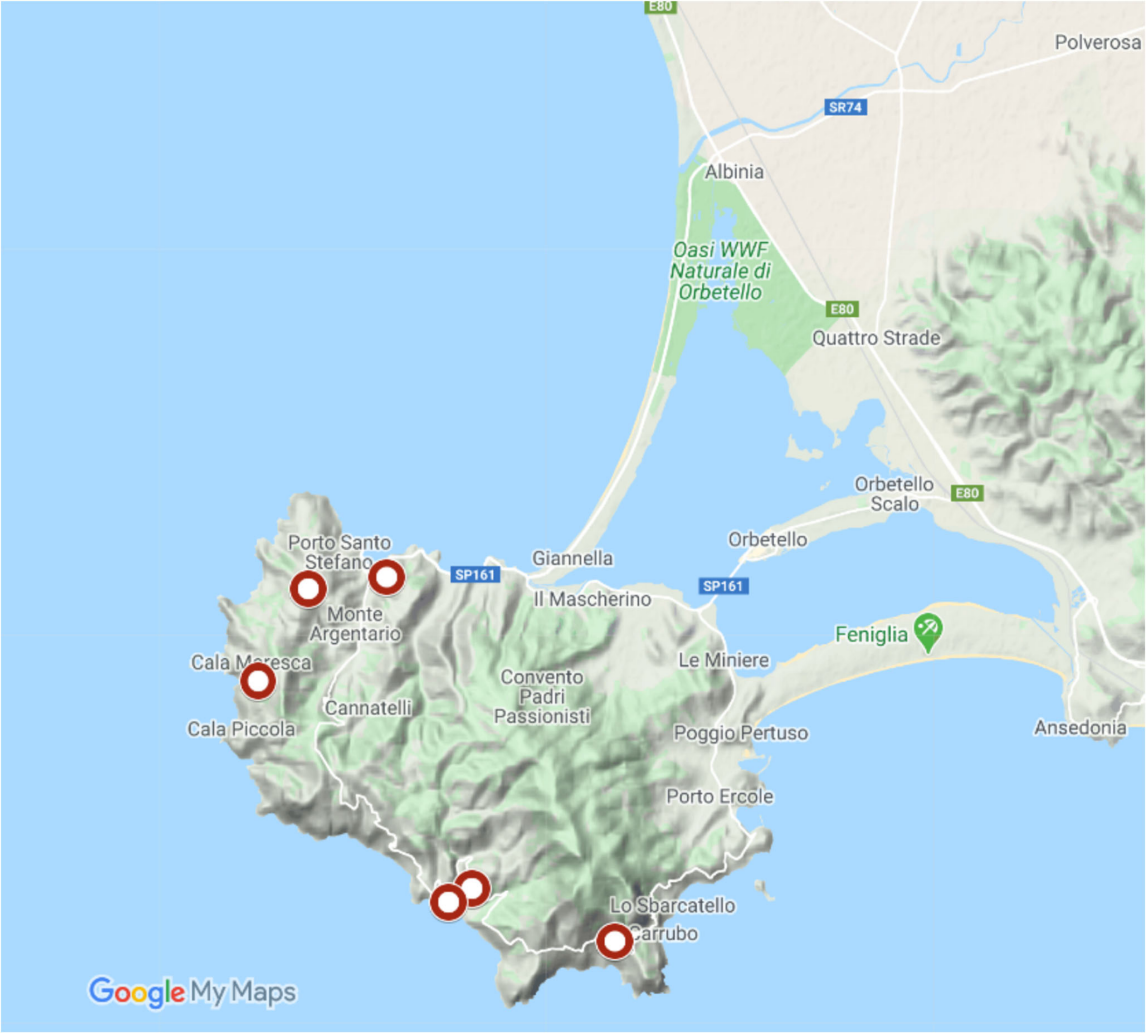
Map of the Monte Argentario promontory (Grosseto, Tuscany, Italy). Dark red circles indicate the position of the *S. junceum* plants from which Xfm ST87 was successfully isolated

After the second round of purification, single colonies on BCYE agar were of an opalescent white color, mucoid when touched with a loop, slightly convex, and circular with entire margins with a diameter of 1 mm after 30 days of incubation at 28 °C (Fig. 1b). Nearly all isolates (110 out of 116) displaying this colony morphotype tested positive for Xf according to Harper et al. (2010). Based on these results, Xf was successfully isolated from 6 out of 11 plants of *S. junceum*, each sampled in a different location of Monte Argentario (Fig. 2)*.*

**Fig. 3:**
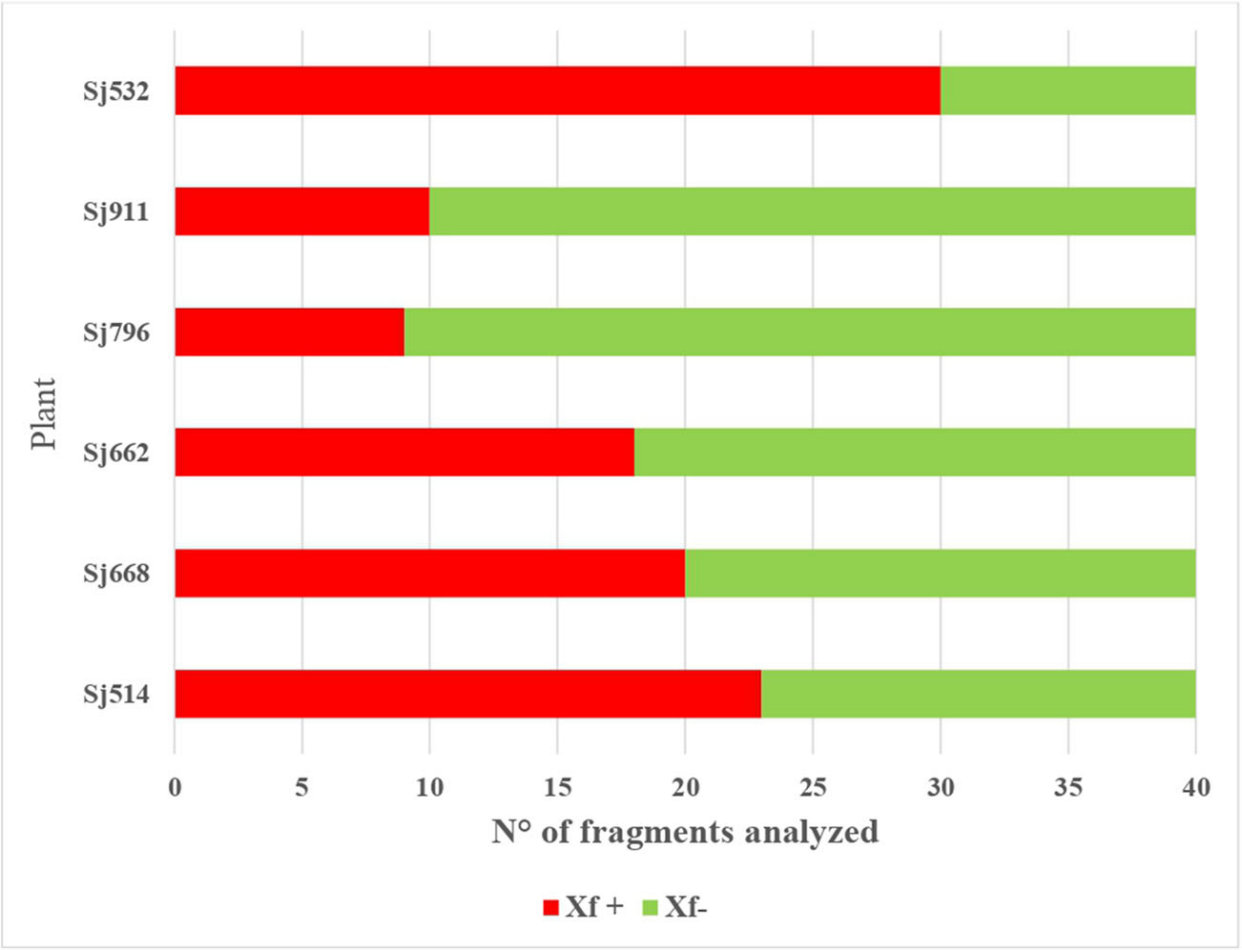
Absolute frequencies of isolation of Xf from 6 plants of *S. junceum*. From each plant, 40 green shoot fragments were chosen, squeezed with sterile pliers and the sap from one of the exposed ends blotted onto BCYE agar plates. After 8 weeks of incubation at 28 °C, the number of sap blots from which Xf was successfully isolated based on the results of qPCR (Harper et al. 2010) was counted

The frequency of Xf isolation from fragments of these six plants ranged from 22 to 75% (Fig. 3). Two isolates from each plant (12 isolates in total) were arbitrarily selected and were confirmed to belong to ST87 [5 (*leuA*), 3 (*petC*), 5 (*malF*), 3 (*cysG*), 3 (*holC*), 21 (*nuoL*), 3 (*gltT*)] by MLST-typing analysis.

Based on these findings, there is circumstantial evidence that Xfm ST87 is the most recurrent if not sole *X. fastidiosa* phylotype in Monte Argentario.

### Immunohistochemistry analysis

**Fig. 4:**
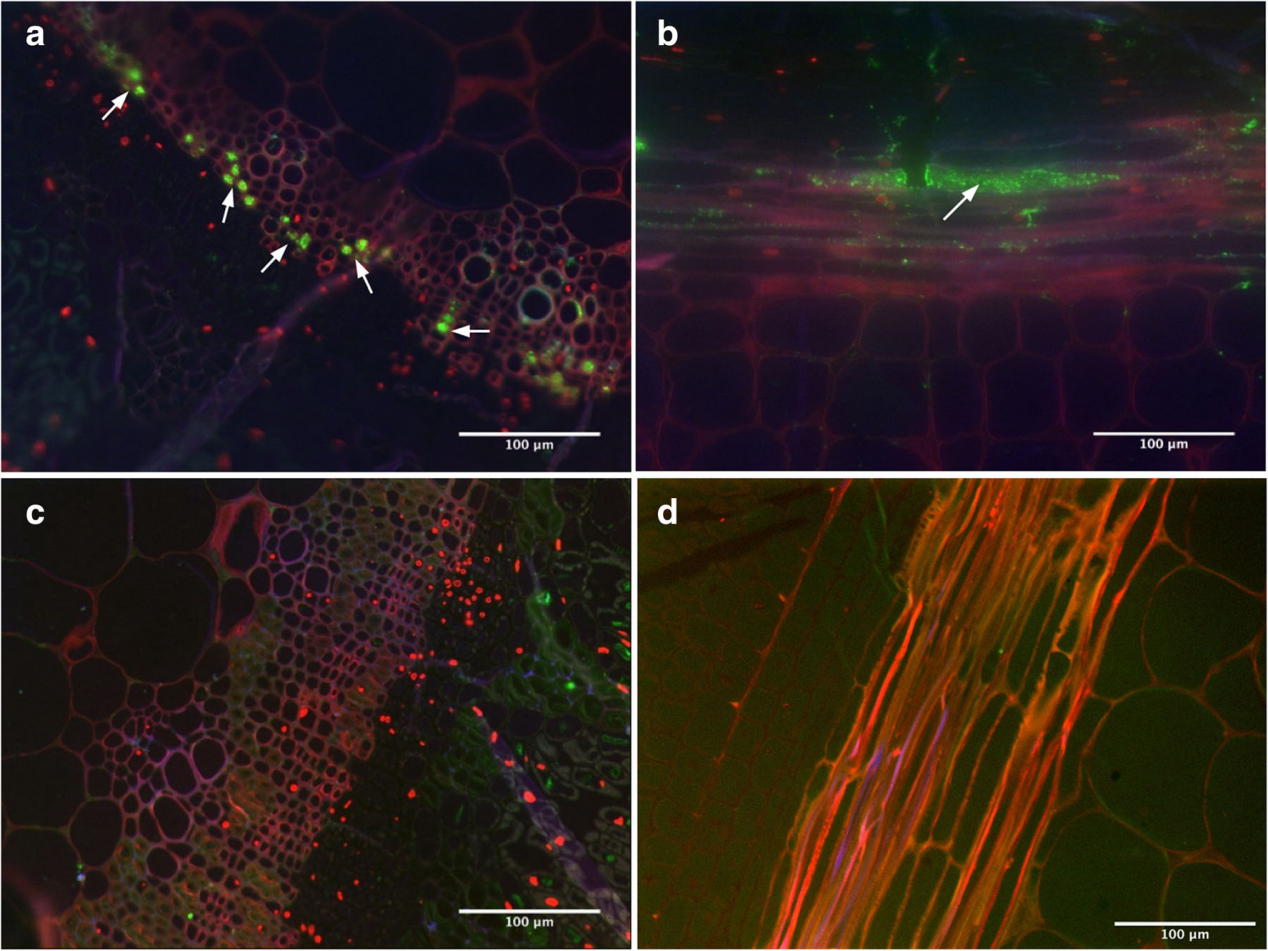
Fluorescence images of cross (**a**, **c**) and longitudinal (**b**, **d**) sections of primary shoot of SJ 668 and control plant after immunohistochemical staining. **a** Bacteria localized in xylem vessels close to the cambium (white arrows); **b** bacteria localized in lignified elements (white arrow); **c** and **d** negative control

The presence of Xfm ST87 in infected plants was confirmed by immunohistochemical analysis. Bacterial cells were observed in several xylem vessels (Fig. 4a), close to the cambium tissue in cross and longitudinal sections where bacteria fully colonized lignified elements (Fig. 4b). In healthy plants, the presence of the bacterium in the xylem vessels of primary shoots was never detected in either the transverse (Fig. 4c) or longitudinal section (Fig. 4d).

### Anatomical observations

**Fig. 5:**
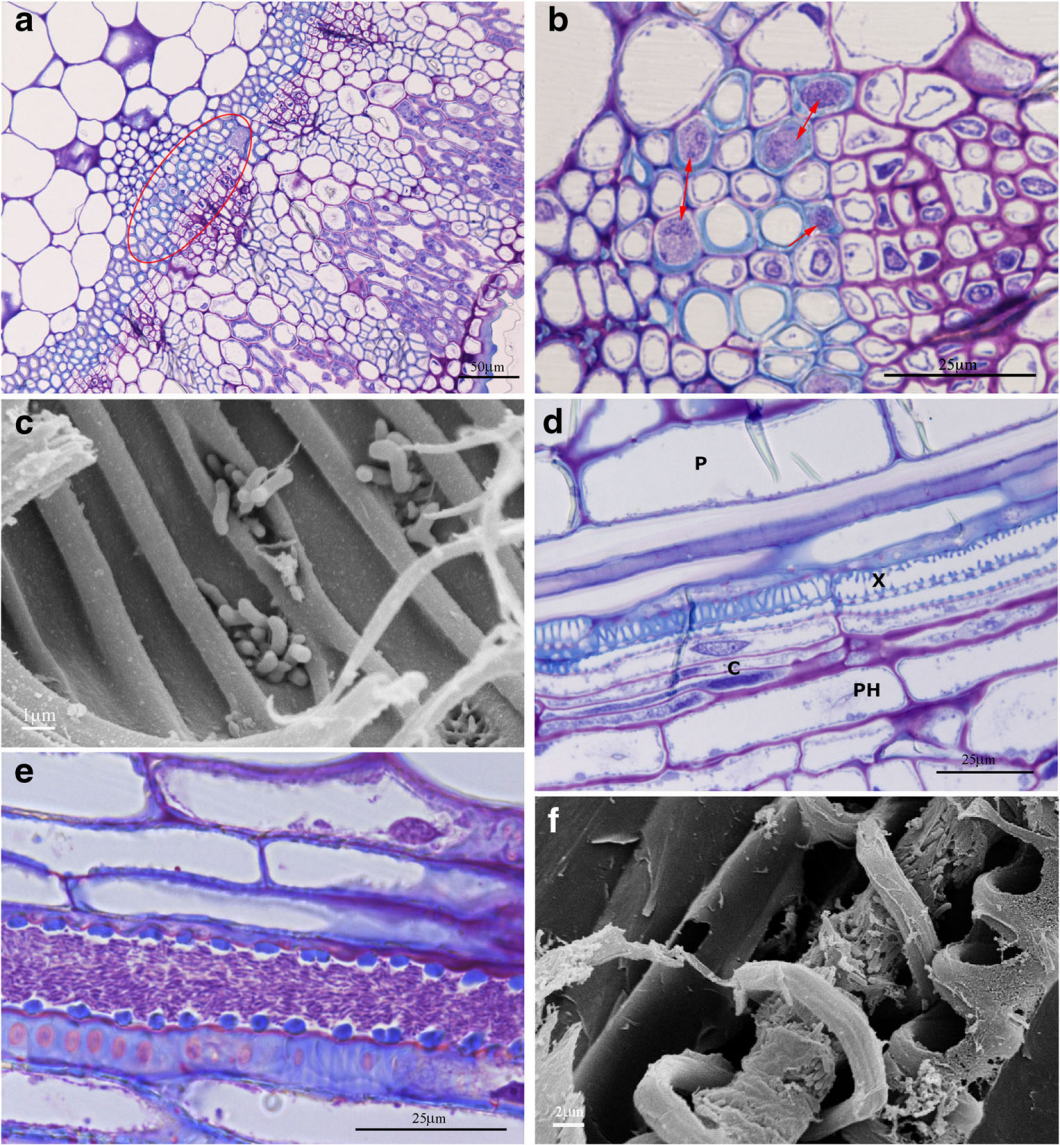
LM images of transversal (**a**, **b**) and longitudinal (**d**, **e**) sections of young shoots of SJ 668 stained with Toluidine blue. SEM images (**c**, **f**) of longitudinal sections of young shoots of SJ 668. **a** Presence of a bacterial colony (surrounded by red circle) close to a vascular bundle. **b** Magnified detail of image (**a**): several cells with lignified walls are occupied by bacterial colonies (red arrows). **c** Bacteria crossing the pits of the adjacent vessel. **d** Healthy shoot tissue (negative control) where the phloem (PH), cambium (C), xylem (X), and pith (P) are visible. **e** Vessel infected by Xfm ST87. **f** SEM image of the longitudinal section of a young shoot showing bacteria filling a vessel

LM images captured from cross and longitudinal sections of green shoots of SJ 668 samples are shown in Fig. 5. Xfm ST87 was observed in a group of xylem vessels by the previous cribro-vascular bundle (Fig. 5a). In particular, Fig. 5b shows the presence of several neighbor vessels colonized by bacteria (indicated by red arrows). It seems that bacteria horizontally infected adjacent vessels moving through the pits that connect the whole vascular system as previously observed in *Olea europea* L. petioles colonized by *X. fastidiosa* subsp. *pauca* ST53 (Cardinale et al. 2018; Novelli et al. 2019). This diffusion mechanism was also confirmed by the SEM micrograph shown in Fig. 5c where bacteria were found crossing the pits. Another observation is that bacteria were never found in the living cells of cambium or phloem tissues, supporting the hypothesis that Xfm ST87 largely survives surrounded by dead cells throughout its life. This phenomenon was also observed in green shoots of SJ 911 (Fig. SI 1 A-B) and SJ 796 (Fig. SI 2 E).

In *S. junceum* twigs, healthy tissues showed key features of the primary structure as shown in Fig. 5d, where no bacterial cells were observed in xylem conductive elements. Other examples of healthy organs (i.e., the ovary, the twig, and the leaf) of *S.junceum*, in particular of SJ 777, are shown in Fig. SI 2 (A-D). Interestingly, bacteria occupying the conductive element were found arranged parallel to each other following the watercourse, until they totally occluded the vessel (Fig. 5e). This was confirmed in a SEM micrograph where a vessel with spiral wall lignification was obstructed by the high density of bacteria in the lumen (Fig. 5f).

**Fig. 6:**
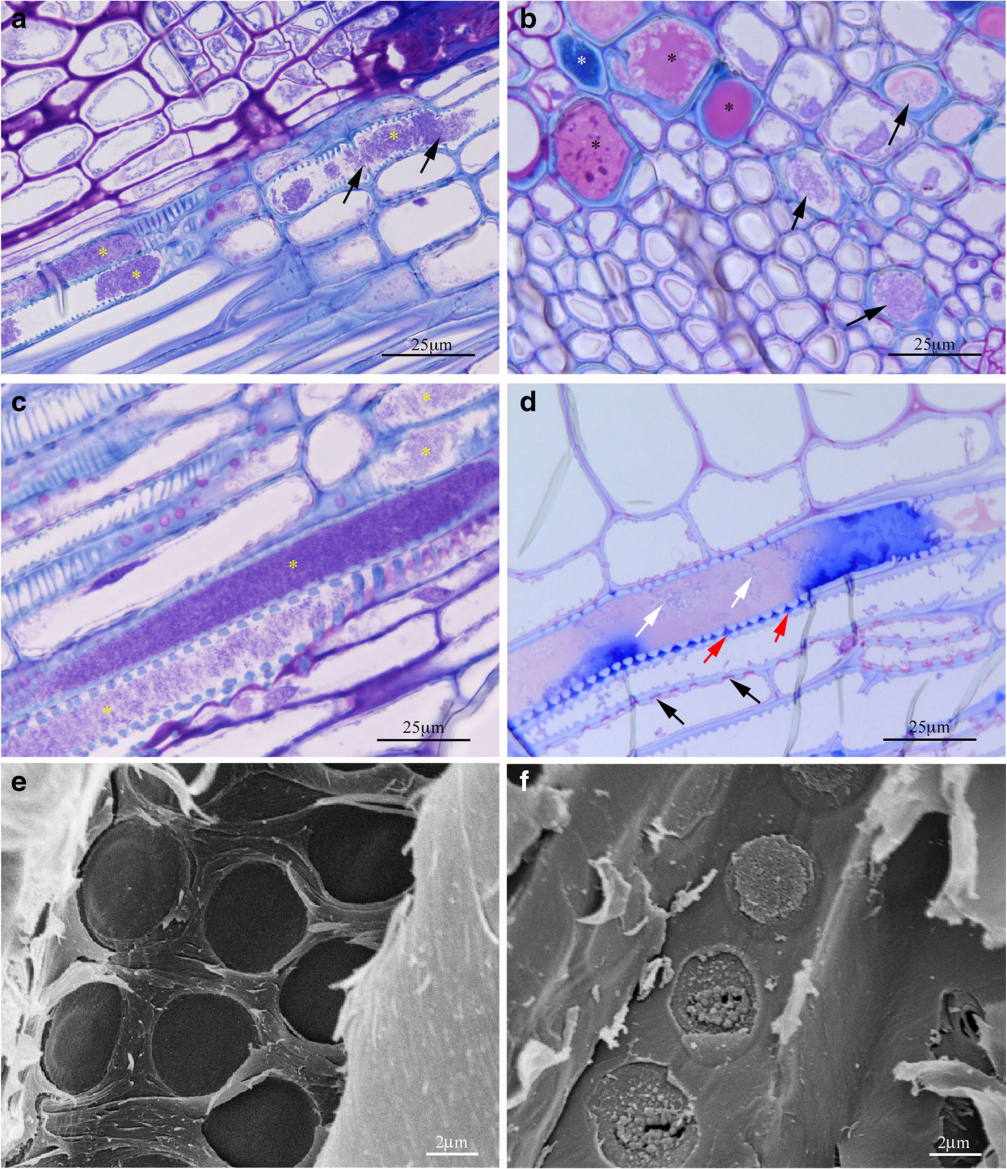
LM images obtained from longitudinal (**a**, **c**, **d**) and cross (**b**) sections of a twig of SJ 668 stained with Toluidine blue. SEM images (**e**, **f**) of longitudinal sections of young shoots of SJ 668. **a** Vessels of young shoot infected by Xfm ST87 at an early stage. Bacterial colonies (asterisks) occupy part of the vessel lumen crossing perforation plates (black arrows). **b** Cross-section showing bacteria in the lumen of several vessels colonized by bacteria (black arrows), the presence of pink/violet stained mucilage in tracheary elements (black stars) in one of which degenerated bacteria are observed, and a blue-stained matrix (white star). **c** Vessels infected by Xfm ST87 at a later stage. Bacterial colonies (asterisks) completely occupy some of the vessels’ lumen. **d** A conductive element with two stainings: blue and pink. A darker blue staining was observed in the pits (red arrows) compared to the pits of unaffected vessels (black arrows). Moreover, white arrows indicate damaged or dead bacterial cells in the pink matrix. **e** Negative control of pits. **f** SEM micrograph showing a granular matrix corresponding to the blue gel occluding the pits as shown in Fig. 5d

In Fig. 6a, the longitudinal section of older shoots shows intermediate infection stages up to vascular plugging. At the early stage of colonization, bacteria interacted with the xylem wall probably using adhesins, which have been reported to be produced by Xf in large amounts in comparison to other bacteria (Simpson et al. 2000; Van Sluys et al. 2003). Then, bacterial cells were joined by more cells and the diffusion process initiated, first colonizing the single vessel element through its perforation plates and, subsequently spreading in the proximity, digesting the pit membranes of adjacent xylem cells (Newman et al. 2003; Roper et al. 2019). It seems that the interaction between Xfm ST87 and the host is not univocal. Once in the xylem vessels, Xfm ST87 cells adhere to the plant cell wall as well as to each other, coordinating the exploratory movement along the conduits to enhance disease progression, similarly to what is reported by Novelli et al. (2019) for Xfp ST53 in olive. At the same time, the host plant recognizes an exogenous presence and responds to the pathogen with physical and chemical post-infection barriers. In our opinion, this plant defense response is shown in Fig. 6b where the presence of bacteria is associated with a pink/red stained matrix which is probably related to gels (gum) secretion by the parenchyma cells (Rioux et al. 1998). The bacteria appear trapped/concentrated in the matrix, where they self-replicated filling at a later stage the entire vessels (about 450 μm long), thus occluding the water passage (Fig. 6c).

The chemical nature of the gels inside the xylem vessels was heterogeneous. The pink/violet Toluidine blue staining (identified in Fig. 6b by black stars) would indicate the presence of gels (mucilage) in the vessels, as previously observed using specific dyes and thorough extractions (Dong et al. 1997; Tagne et al. 2002; Crews et al. 2003), indicating a high content of acidic polysaccharides. The pink-staining matrix was observed when bacteria were absent or dead, suggesting that it was produced by *S. junceum* as a defense response against their spreading (Fig. 6b). Amounts of pink-staining material with Toluidine blue were found in cotyledons of *Brassica napus* infected by *Leptosphaeria maculans* (Roussel et al. 1999). In addition to the pink-staining matrix, blue-staining phenolic materials were also observed in maize vessels following mechanical wounding, as reported by Crews et al. (2003). The two different color reactions were also observed in SJ 911 green shoot sections (Fig. SI 1 C and D). Furthermore, in Fig. 6d, a particular vessel element showed both colors, blue and pink/violet. The dark blue-stained mucilage was also found at higher concentrations in the pits and inter-vessels compared to those of conduits noninfected conduits (black arrows). We observed that bacterial cells found in the presence of the pink-stained region appeared degenerated compared to bacteria shown in Fig. 5c. For this reason, we hypothesized that they might be damaged or dead (shown by white arrows). In comparison with the negative control shown in Fig. 6e, the blue gel can be associated with a granular matrix that apparently caused the formation of pit plugs, as shown in Fig. 6f. Since the xylem vessel is not able to produce mucilage, the surrounding parenchyma cells must be involved in the task. The blockade of the pits by granular plugs may reduce the capability of the bacteria to move from a xylem column to the adjacent ones.

**Fig. 7:**
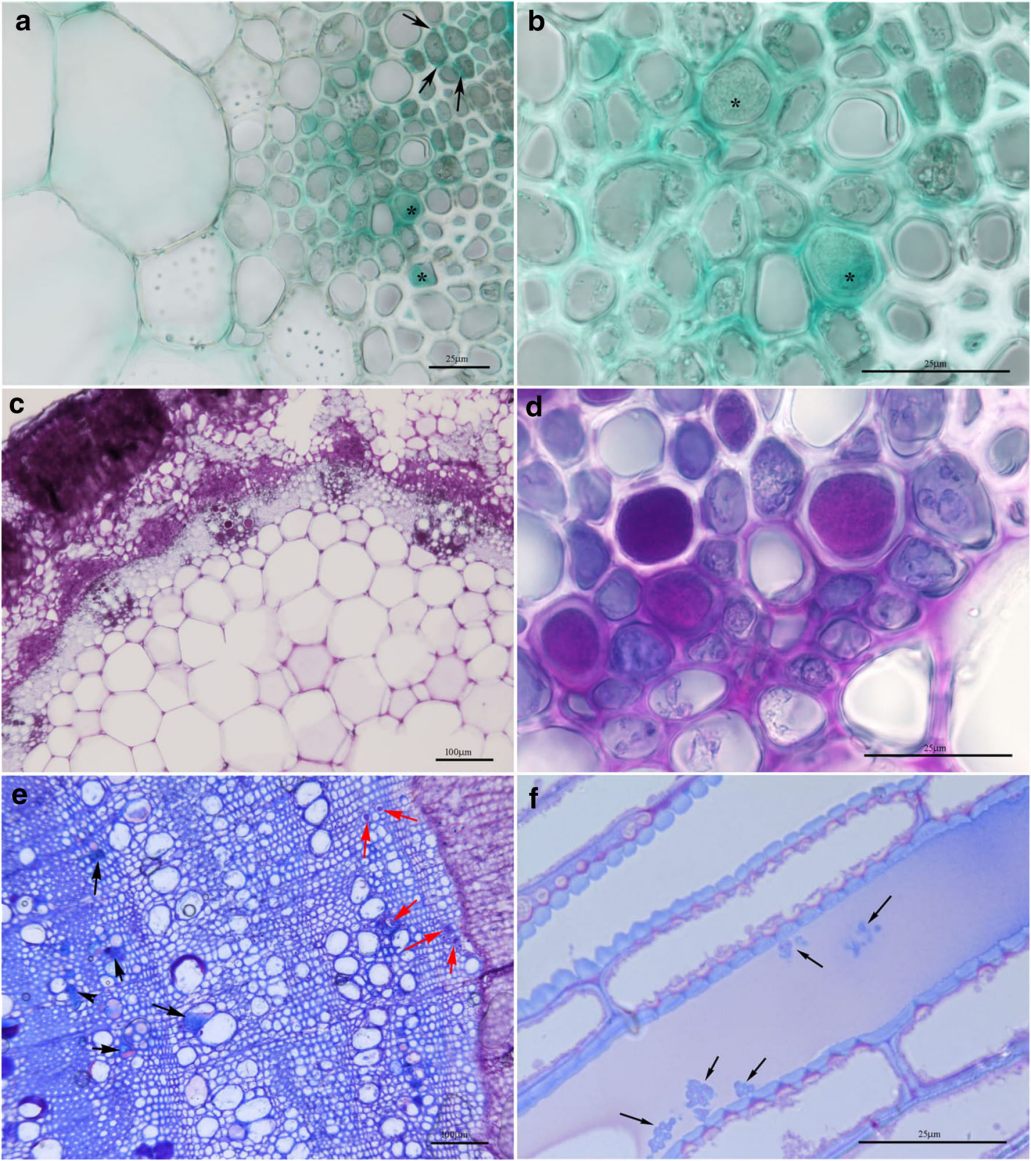
LM images of SJ 668 samples. **a** Transverse sections of a twig region stained with Alcian Blue for acidic mucopolysaccharides in the pits (black arrows) and in the vessels lumen (asterisks). **b** Detail of (**a**) in which the presence of mucopolysaccharides (asterisks) is found. **c** Transverse section of a twig stained with PAS reaction for polysaccharides. **d** Detail of (**c**) in which bacteria are found in the presence of polysaccharides. **e** Transverse section of the older branch stained with Toluidine blue. Bacteria are indicated by red arrows, polysaccharide and phenolic components by black arrows. **f** Longitudinal section of a green shoot stained with Toluidine blue. Bacteria are apparently covered by a blue layer (arrows)

The nature of the gel composition was clarified using two different stainings, Alcian Blue (Fig. 7a and b) and PAS reaction (Fig. 7c and d), which are specific for acidic mucopolysaccharides and polysaccharides, respectively.

Then, the presence of Xfm ST87 was investigated in older branches (Fig. 7e) in order to localize the bacteria in the different growth rings. From the transverse section, it was possible to date the age of the shoot, which was approximately 3 years. Bacteria were found in the cell lumen of the younger growth ring but not observed in the oldest. The vessel elements in the oldest growth ring were characterized mainly by the presence of polysaccharide and phenolic matrices without any bacteria, once again confirming that the presence of polysaccharides and phenolic gels in the lumen are not always associated with bacteria.

In some vessels, bacteria were coated by a thin blue layer and the blue matrix corresponding to the phenolic component was observed around few bacteria (Fig. 7f).

Bacteria were observed in SJ 796’s leaf sections (Fig. SI 1 E) while flower sections of SJ 796 and root sections of SJ 668 did not show any bacterial colonization (Fig. SI 1 F, 1 G, and 1 H).

The results presented in this paper indicate that a single phylotype of Xf subsp. *multiplex* (Xfm), i.e., ST87, is infecting *S. junceum* plants growing in the demarcated area of Monte Argentario (Grosseto, Tuscany, Italy). This finding, along with the fact that ST87 has been previously reported from other hosts growing in the same area and that draft genomes of three ST87 strains have an average nucleotide identity of 99.99%, brings compelling evidence of a widely spread phylotype, if not sole, of Xf at Monte Argentario (Marchi et al. 2018; Saponari et al. 2019; Giampetruzzi et al. 2019).

After the first record in the European Union territory on olive (Saponari et al. 2013), the presence of Xf subspecies *pauca*, *multiplex*, and *fastidiosa* was recorded in cultivated as well as in spontaneous Mediterranean plant species growing in natural and urban landscapes of Italy, France, Spain, and Portugal. Some of these plants were previously unreported hosts of these Xf subspecies, and many of them were found to be infected by different STs of different Xf subspecies in different countries and locations (EPPO 2020). Apart from underlining once more the ability of Xf to become rapidly adapted to new environments, the European scenario also indicates the existence of hosts whose potential role as inoculum sources in current and future Xf outbreaks needs to be rapidly addressed. Some of them are common plants of commerce, as in the case of the ornamental plants *Polygala myrtifolia* and *Lavandula dentata*, or of fruit trees like *Prunus dulcis* (EC, 2019). Others, as for example *Calicotome spinosa*, are present in shrubland areas throughout the Mediterranean basin. Finally, plants like *Rosmarinus officinalis* and *Cistus albidus* as well as *S. junceum* itself not only grow spontaneously but are also cultivated as ornamentals in gardens and parks. Despite these findings, to the best of our knowledge, the anatomy of olive plants naturally infected by Xf subsp. *pauca* ST 53 is the only one that has been studied and characterized in Europe to date (De Benedictis et al. 2017; Cardinale et al. 2018; Sabella et al. 2018; Sabella et al. 2019).

**Fig. 8:**
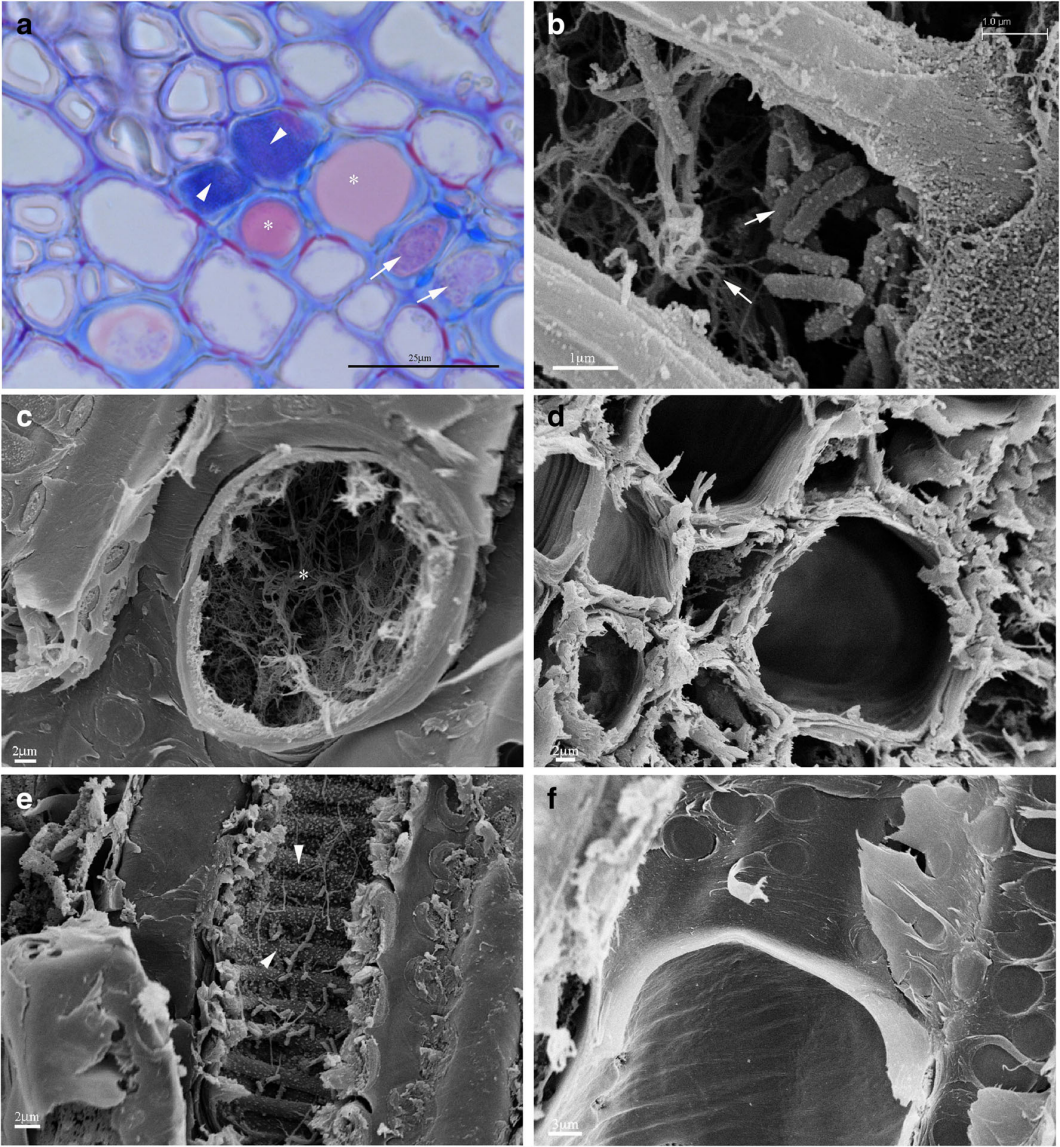
LM image (**a**) and SEM micrographs (**b**, **c**, **d**, **e**, and **f**) of SJ 668. **a** Cross-section of a green shoot with polysaccharide (pink/violet indicated by asterisk) and phenolic (blue indicated by triangle) matrices stained with Toluidine blue. Vessels colonized by bacteria (arrows). **b** Cross-section (corresponding to **a**) of a twig where xylem vessels were colonized by Xfm ST87 in association with filamentous (red arrow) and granular (yellow arrow) matrices. **c** Cross-section of a xylem vessel showing a filamentous matrix (asterisk). No bacteria are visible. **d** Cross-section of the negative control green shoot showing xylem vessels without any bacteria. **e** Longitudinal section where granular matrices (triangle) were identified in the vessel lumen. **f** Longitudinal section of the negative control green shoot without any bacteria

As a typical Mediterranean species, *S. junceum* presents morphological features, such as ephemeral leaves and green-photosynthetic stems that make it perfectly adapted to a xerophytic environment (Bezić et al. 2003; Nilsen et al. 1993). Hence, the anatomy of young shoots is characterized by photosynthetic cortical parenchyma with crystals, intercalated by groups of sclerenchyma fibers below a multi-layered epidermis with a thick cuticle. The secondary xylem presents distinct growth ring boundaries with a porous or semi-porous wood, vessels in a dendritic pattern, and the axial parenchyma represented by paratracheal parenchyma. It is well known that the possible plant defense responses against pathogens are at the anatomical level, where some specific features such as gels or tyloses allow to compartmentalize the spread of the pathogen. Studies on anatomical plant responses induced by pathogens have been published since the 1980s. In particular, when studying interactions between tomato and *Verticillum albo-atrum*, Street et al. (1986) concluded that the pathogen could induce the production of two distinct coating forms that infused pit membranes and primary walls or lined the secondary walls of xylem vessels. Working with woody plants native to Switzerland, Bonsen and Bonsen and Kučera (1990) observed a correlation between the minimum pit aperture diameter (3 μm) of a vessel-parenchyma pit pair and the type of vessel occlusion by tyloses or gums: below that minimum, gum production occurred, while above 3 μm, tyloses were observed. In our LM observations, we found a narrower pit aperture diameter (< 3 μm) which may justify the presence of gels (gums) in infected tracheary elements. Despite the presence of a paratracheal parenchyma, tyloses such as those observed in naturally infected plants of olive and *Vitis vinifera* (Cardinale et al. 2018; Roper et al. 2019) were never found in any of the infected *S. junceum* samples. Artificial infection with Xf induced tyloses in *V. vinifera* and *V. smalliana* but not in *V. arizonica/candican*, which produced fibrillary networks and gum occlusions (Fritschi et al. 2008). In our LM observations, the polysaccharides nets and the phenolic components were recognized through Toluidine blue staining, resulting in pink/violet and blue coloration, respectively (Fig. 8a). Thus, it seems that these polysaccharidic and phenolic components appear to be related to two different morphologies: fibrillary and granular, respectively, as observed in SEM micrographs (Fig. 8b). Furthermore, by observing Fig. 8b, we hypothesized that the thin layer coating the bacteria in Fig. 7f showed a granular matrix. Instead, filamentous networks were observed in the lumen of xylem vessels of twigs’ transverse sections (Fig. 8c), often associated with the presence of bacteria and a granular matrix, and which were absent in the negative control (Fig. 8d). Similar to our observations in *S. junceum*, Alves et al. (2009) found a sequential process of xylem vessels occlusion in *Citrus sinensis* leaf petiole and blade naturally infected by Xf, where, following the first steps of colonization, bacterial cells became covered by a fibrillary material until completely occluding the vessel lumen. Moreover, the granular matrix was observed filling the vascular lumen of the xylem vessel (Fig. 8e) in contrast to the negative control (Fig. 8f).

## Conclusions

In this study, the presence of Xfm ST87 was detected by PCR and immunohistochemistry, in the xylem cells of naturally infected *S. junceum* plants in the outbreak area of Monte Argentario (Grosseto, Tuscany, Italy).

LM and SEM observations showed that bacteria were able to completely fill the xylem vessels after some intermediate steps of infection and spread through the pits in the adjacent conductive elements.

The host was apparently able to react to the infection by producing gums/mucilage and phenols. Xylem vessels containing a high amount of mucilage did not show presence of bacteria, while SEM images showed granular material adhering to bacterial cells, which was probably produced by the surrounding xylem parenchyma. These mechanisms may be considered as an example of constitutive defense systems of the plant against xylem pathogens. Interestingly, bacteria were also found in the conductive elements of the leaves of one of the naturally infected *S. junceum* plants subject of this study (SJ 796), but they were not found in either the root system or in the flowers.

Artificial inoculations will allow to depict the time course of Xfm ST87 host colonization as well as that of *S. junceum* responses to the invading pathogen.

## Supplementary Information

ESM 1 (PDF 1291 kb)
