# Supplementary material for: Anatomical and biochemical studies of Spartium junceum infected by Xylella fastidiosa subsp. multiplex ST 87
Source: Protoplasma. 2021 Apr 15;259(1):103–15. doi: 10.1007/s00709-021-01640-2 (PMC8752565; doi:10.1007/s00709-021-01640-2)
Supplement: Supplementary file 1 — (PDF 1291 kb) [file 709_2021_1640_MOESM1_ESM.pdf]

Supporting Information

Anatomical and biochemical studies of *Spartium junceum* infected by *Xylella fastidiosa* subsp. *multiplex* ST 87

S. Falsini[1], C. Tani[1], G. Sambuco[1], A. Papini[1], F. Ranaldi[2], S. Campigli[3], L. Ghelardini[3], G. Bleve[4], D. Rizzo[5], M. Ricciolini[5], I. Scarpelli[5], L. Drosera[5], P. Faraoni[2], F. Peduto Hand[6],, G. Marchi[3], and S. Schiff[1]

[1] Dipartimento di Biologia, Università degli studi di Firenze, via P.A. Micheli 3, 50121, Firenze, Italy;

[2] Dipartimento di Scienze Biomediche, Sperimentali e Cliniche, Università degli Studi di Firenze, viale G.B. Morgagni 50, 50134, Firenze, Italy;

[3] Dipartimento di Scienze delle Produzioni Agroalimentari e dell'Ambiente, Università degli Studi di Firenze, Piazzale delle Cascine 28, 50100 Firenze, Italy; [4] Istituto di Scienze delle Produzioni Alimentari, Consiglio Nazionale delle Ricerche, Lecce, Italy;

[5]Regione Toscana, Servizio Fitosanitario Regionale e di Vigilanza e Controllo Agroforestale, Via A. Manzoni 16, 50121 Firenze, Italy.

[6] Department of Plant Pathology, The Ohio State University, Columbus, OH 43220, USA.

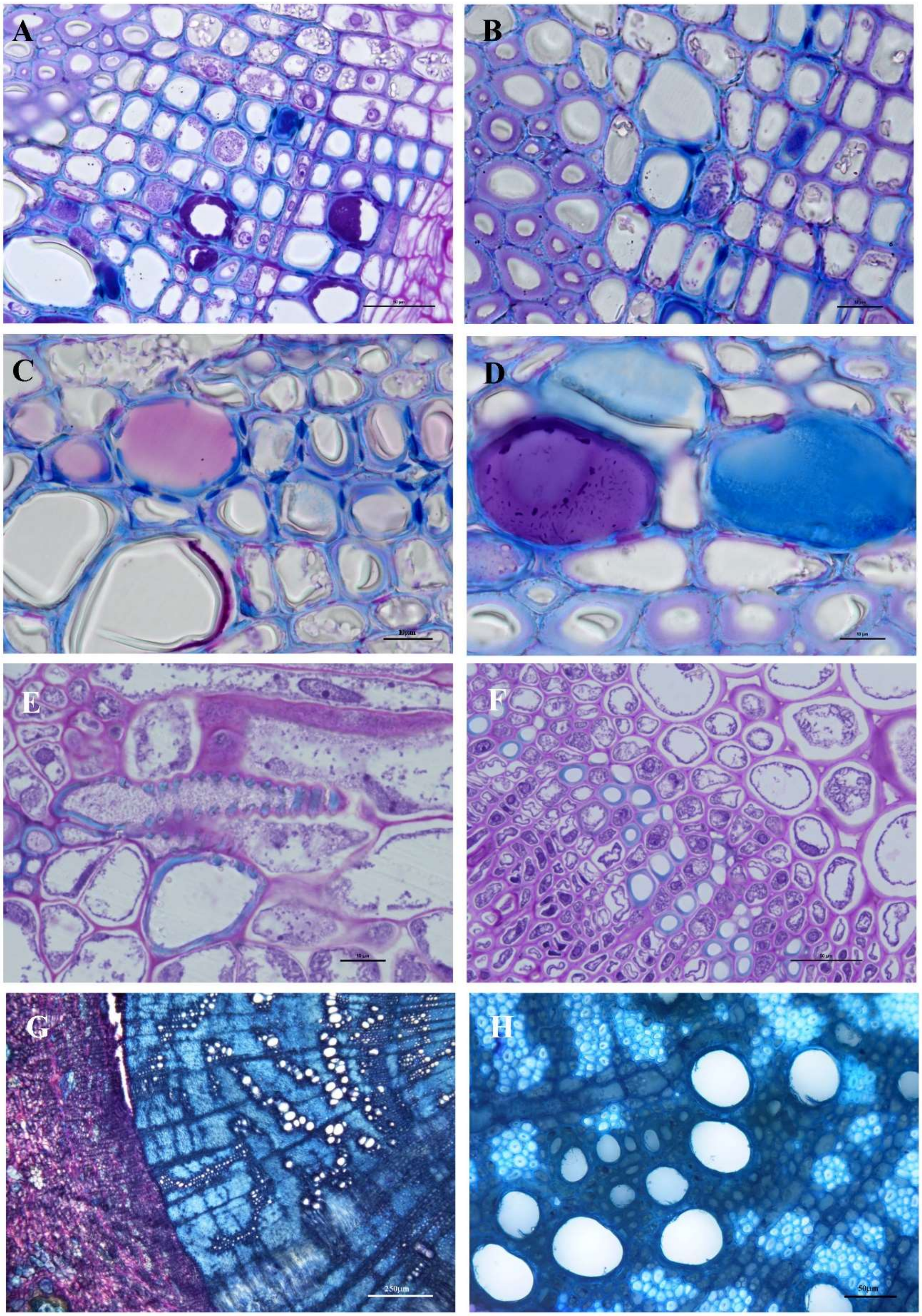

Figure SI 1: Cross sections (A, B, C and D) of SJ 911's twig. The presence of Xf is evidenced in A) and B). C) shows the pink/violet matrix and the blue gel is evidenced in D). Cross sections of the leaf and the flower (E and F) obtained from SJ 796. E) Bacteria in the leaf's xylem vessel; F) No bacteria are evidenced in the vessels. G) and H) Cross

sections of SJ 668's root. G) No bacteria are in the conductive elements. H) Detail of image G). All sections are stained with Toluidine blue.

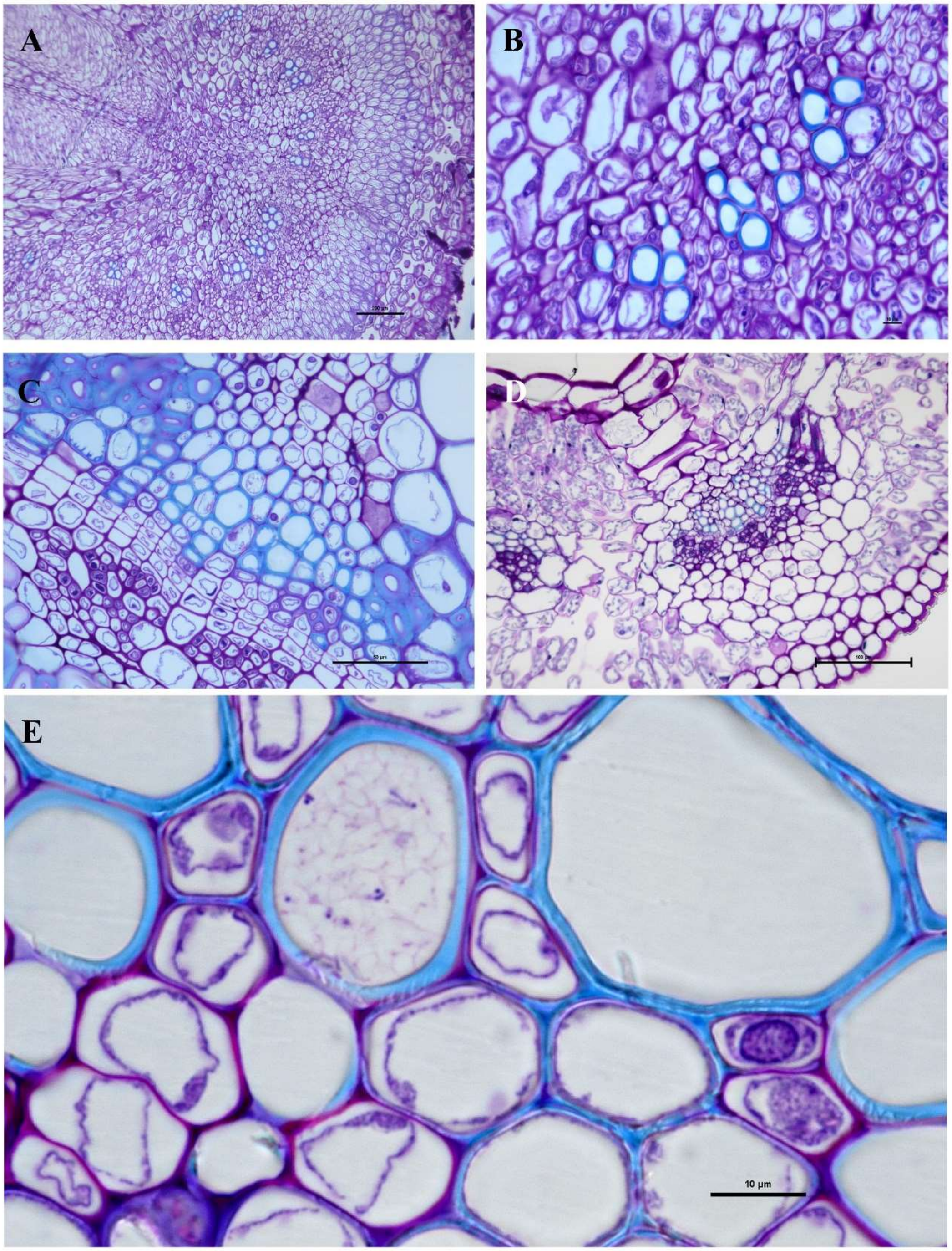

Figure SI 2: Cross sections of SJ 777's different anatomical parts: the ovary (A and B), the twig (C) and the leaf (D). No bacteria are evidenced. E) Cross section of SJ 796's twig. Bacteria in the xylem vessel.
